# S-9-PAHSA ameliorates cognitive decline in a type 2 diabetes mouse model by inhibiting oxidative stress and apoptosis via CAIII modulation

**DOI:** 10.3389/fnmol.2025.1617543

**Published:** 2025-08-08

**Authors:** Xin-Ru Wang, Shan-Shan Huang, Meng Wang, Jin-Hong Lin, Jian-Tao Wang, Jiao-Qi Ren, Cheng-Feng He, Wen-Jiao Xue, Yin Wang, Xue-Chun Wang, Yan-Li Zhang, Ji-Chang Xiao, Jing-Chun Guo, Hou-Guang Zhou

**Affiliations:** ^1^Department of Geriatric Neurology of Huashan Hospital, National Clinical Research Center for Aging and Medicine, Fudan University, Shanghai, China; ^2^Department of Translational Neuroscience, Jing’an District Centre Hospital of Shanghai, State Key Laboratory of Medical Neurobiology and MOE Frontiers Center for Brain Science, and Institutes of Brain Science, Fudan University, Shanghai, China; ^3^Department of Geriatric Medicine, Qilu Hospital of Shandong University, Jinan, Shandong, China; ^4^Key Laboratory of Organofluorine Chemistry, Shanghai Institute of Organic Chemistry, University of Chinese Academy of Sciences, Chinese Academy of Sciences, Shanghai, China

**Keywords:** S-9-PAHSA, diabetes-associated cognitive disorder, oxidative stress, mitochondrial dysfunction, CAIII

## Abstract

**Purpose:**

S-palmitic acid-9-hydroxy stearic acid (SP), a newly characterized endogenous lipid with multifaceted biological activities, is poised to shed light on its potential in diabetes-related cognitive disorder (DRCD). This study aims to uncover the effects of SP on DRCD and the underlying mechanisms.

**Methods:**

C57BL/6 mice were fed with high-fat diet for 5 months to induce type 2 diabetes mellitus (T2DM). Subsequently, they received bilateral hippocampal injections of adeno-associated virus (AAV) carrying carbonic anhydrase III (CAIII) shRNA or control shRNA. Following one-month treatment with SP or vehicle, cognitive function was assessed using the Morris water maze and Y-maze tests. Oxidative stress and apoptosis were measured by Enzyme-linked Immunosorbent Assay (ELISA), and hippocampal neuronal morphology was examined through HE, Nissl, or NeuN staining. RNA sequencing (RNA seq), cell viability, tetramethylrhodamine ethyl ester (TMRE) staining, and mitoSOX assays were also performed in cultured PC12 cells.

**Results:**

Our findings demonstrated that CAIII played a pivotal role in enhancing cognitive function in T2DM mice by improving spatial memory. SP ameliorated hippocampal injury by CAIII-mediated AMPK/Sirt1/PGC1α pathway, Bcl-2/Bax ratio elevation, and cleaved-Caspase 3 reduction. CAIII participated in various biological processes in the effects of SP on PC12 cells, including cell viability, lactate dehydrogenase (LDH) release, antioxidant enzymes, the maintenance of mitochondrial membrane potential, and the reduction of mitochondrial reactive oxygen species (ROS).

**Conclusion:**

Our study revealed that CAIII was integral to the effects of SP on DRCD, suggesting its potential as a therapeutic target for DRCD.

## 1 Introduction

Type 2 diabetes mellitus (T2DM) is a chronic metabolic disorder characterized by hyperglycemia. Diabetes-related cognitive disorder (DRCD) is one of the common complications of diabetes that attracts widespread attention (McCrimmon et al., 2012). Evidence indicates that diabetes is an independent risk factor for cognitive impairment, with both prediabetes and diabetes accelerating cognitive deterioration (Shang et al., 2021).

Increasing research suggests that multiple neuropathologic mechanisms may be involved in the development and progression of DRCD (Feinkohl et al., 2015). Inhibition of neurogenesis, electrophysiological deficits, oxidative stress, and neuronal apoptosis induce structural changes and participate in dysfunction in the brains of individuals with diabetes (Gaspar et al., 2016), contributing to cognitive function decline. The subsequent deterioration of patients’ self-care ability adversely affects their quality of life, imposing huge burdens on both families and society. However, no targeted clinical therapies for DRCD have been established yet (Honig et al., 2018).

Palmitic acid esters of hydroxy-stearic acids (PAHSAs), a recently identified class of fatty acids, have demonstrated the capacity to enhance insulin sensitivity and possess anti-inflammatory properties (Aryal et al., 2021). 9-PAHSA is the predominant isomer in serum in both insulin-resistant and insulin-sensitive humans. It is also the most abundant isomer in subcutaneous adipose tissue of humans and mice (Yore et al., 2014). Prior studies have shown that S-9-PAHSA (SP) can enhance glucose-stimulated insulin secretion (GSIS) and glucose uptake (Aryal et al., 2021), suggesting a potential benefit in diabetic complications. The impact of SP on DRCD, however, remains to be elucidated.

Carbonic anhydrases (CAs), a class of zinc-containing metalloproteases, are ubiquitously found in prokaryotes and eukaryotes, with diverse isoforms localized across cellular compartments such as the cell membrane, cytoplasm, and mitochondria (Supuran, 2017; Thiry et al., 2007). Carbonic anhydrase III (CAIII), as one of the isoenzymes, is widely expressed in the brain and is highly expressed in the cytoplasm in neurons. It exhibits low catalytic activity for CO_2_ hydration, suggesting additional roles in the brain beyond its catalytic function (Halmi et al., 2006; Supuran, 2015). The role of CAIII in cognitive disorders and its underlying mechanisms warrant further investigation, as current CA inhibitors do not selectively target specific isoforms (Supuran, 2008; Nógrádi et al., 1993). In the present study, *in vivo* and *in vitro* experiments were performed to explore the impacts of SP on DRCD and the potential involvement of CAIII. Exploring the pathogenesis and identifying novel targets for DRCD may pave the way for innovative clinical interventions.

## 2 Materials and methods

### 2.1 Animal and experimental procedure

Male C57BL/6 mice (6–8 weeks old) were procured from Shanghai Lingchang Biotechnology and housed under a controlled temperature (21–23°C) with a 12-h light/dark cycle. All the animal protocols were performed according to the Guide for the Care and Use of Laboratory Animals issued by the National Institutes of Health (NIH) and were approved by the Animal Welfare and Ethics Group of the Department of Laboratory Animal Science of Fudan University (2020-Huashan hospital-JS190).

Cohort 1: Mice (6–8 weeks old) were assigned to either a normal diet (ND, *n* = 15) or high-fat diet (HFD, *n* = 30) for 5 months. Glucose levels were measured, and mice with fasting blood glucose ≥8.0 mmoL/L were classified as T2DM. (Roche, Switzerland). T2DM mice were randomly assigned to either the HFD (*n* = 14) or SP (*n* = 14) group, receiving either a vehicle (50% PEG-400 + 0.5% Tween-80 + 49.5% H_2_O) (Syed et al., 2018) or SP (30 mg/kg/d) in drinking water for 1 month, respectively. The groups were designated as ND, HFD, and SP.

Cohort 2: Mice (6–8 weeks old) were fed either ND (*n* = 15) or HFD (*n* = 80) for 5 months. HFD mice randomly received bilateral hippocampal injections of AAV carrying either CAIII shRNA (HFD + AAV-CAIII sh, *n* = 40) or control shRNA (HFD + AAV-Con, *n* = 40) via stereotaxic surgery. CAIII knockdown was confirmed by real-time PCR and western blotting 3 weeks post-injection. Mice that received either AAV injection were subsequently randomized to receive either vehicle or SP (30 mg/kg/d) in drinking water for 1 month. Body weight, fasting blood glucose, and daily water/food intake were monitored (Supplementary Figure S1).

### 2.2 Fasting blood glucose (FBG), intraperitoneal glucose tolerance test (IPGTT) and intraperitoneal insulin tolerance test (IPITT)

Mice were fasted for 12 h to test fasting blood glucose (FBG). For the intraperitoneal glucose tolerance test (IPGTT), mice underwent a 14-h fast followed by an intraperitoneal injection of 20% glucose (2 mg/kg body weight) in PBS. Blood glucose levels were measured at 0, 30, 60, 90, and 120 min post-injection, and the area under the curve (AUC) was calculated. For the intraperitoneal insulin tolerance test (IPITT), mice were fasted for 5 h before receiving an intraperitoneal insulin injection (1 U/kg body weight, Novo Nordisk, Denmark). Blood glucose was measured at 0, 30, 60, 90, and 120 min after injection, and AUC was calculated.

### 2.3 The synthesis of S-9-PAHSA (SP)

**Figure 1:**
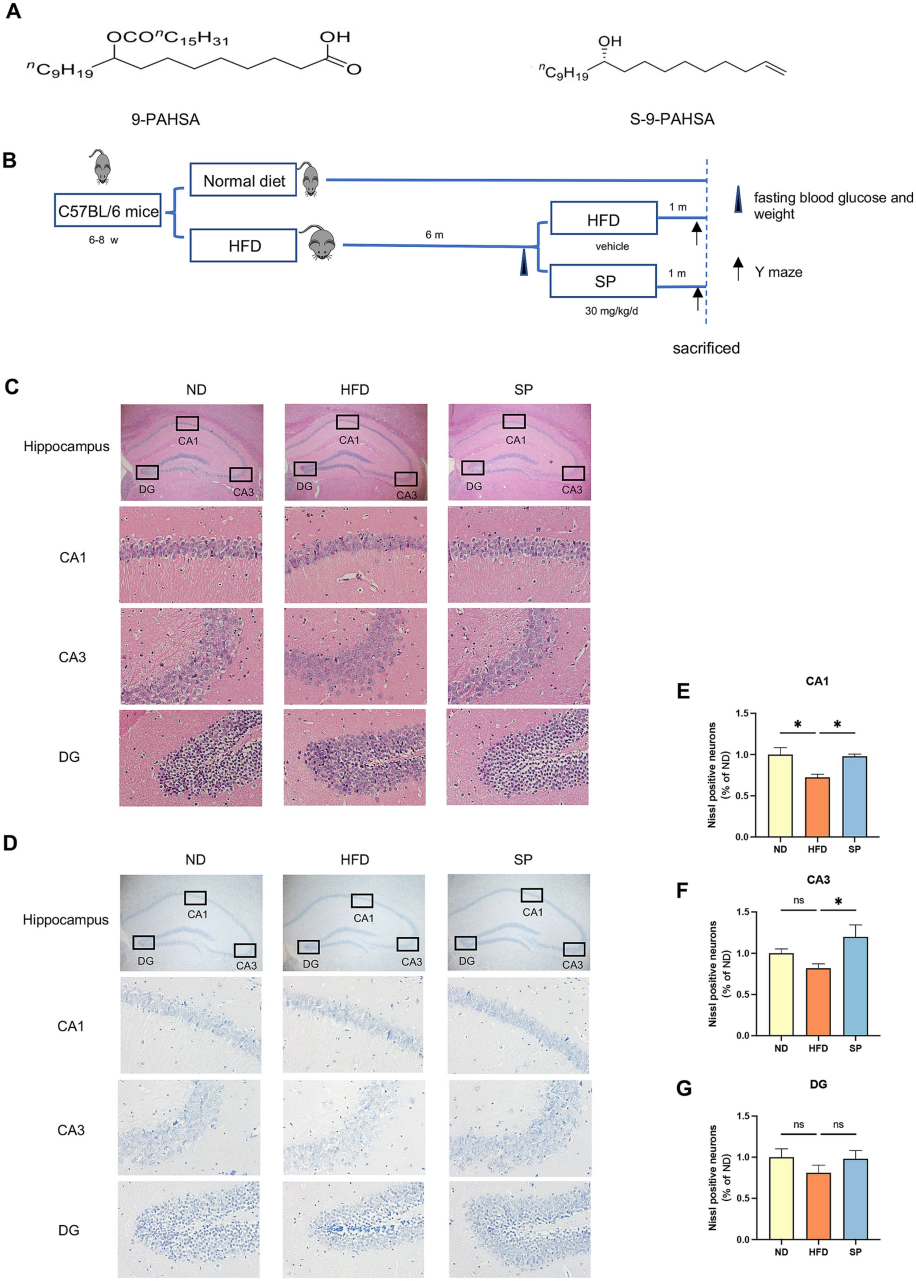
SP ameliorates neuronal damage in the hippocampus of T2DM mice. **(A)** Chemical structures of 9-PAHSA and S-9-PAHSA; **(B)** Schematic illustration of the experimental protocol; **(C)**. Hippocampal HE staining in mice across groups, including whole hippocampus (× 20) and CA1, CA3, and DG regions (× 200), *n* = 4; **(D)** Hippocampal Nissl staining in mice across groups, similarly magnified, *n* = 4; **(E–G)** Mean percentage of Nissl-positive cells in the CA1, CA3, and DG regions, normalized to the ND group; Data are presented as mean ± SEM. *p < 0.05, **p < 0.01, ***p < 0.001, ****p < 0.0001.

SP was synthesized by the Institute of Organic Chemistry, Chinese Academy of Sciences based on a previous study (Nelson et al., 2017). The molecular structures of 9-PAHSA and SP are illustrated in Figure 1A.

### 2.4 Morris water maze (MWM)

The Morris water maze (MWM) was used to evaluate mouse spatial learning and memory. The latency on the training days (d 1 to d 5) was defined as the first time to reach the platform within 60 s. Mice unable to find the platform within 60 s were gently guided to the platform for 15 s. On day 6 (probe trial day), the platform was removed, with trajectories recorded by the video and analyzed by EthoVision software (version 8.5).

### 2.5 Y maze

Y maze was utilized to assess the short-term spatial working memory of mice. Mice were positioned at the end of the designated arm (start arm) and allowed to explore freely for 8 min. The course was recorded via video, with subsequent analysis performed by EthoVision software (version 14.0).

### 2.6 The measurement of SP in tissues

Concentrations of SP in tissues were determined using ultra-high performance liquid chromatography (UPLC-MS/MS). The procedure was conducted by Maixi Medical Technology Co. according to the instructions.

### 2.7 Histology and immunohistochemistry

Mice from four groups (*n* = 4 per group) were perfused with PBS followed by 4% paraformaldehyde. The brains were postfixed in the same fixative overnight at 4°C. After progressively dehydrated, embedded in paraffin, and sliced into 4 μm-thick sections, brain morphology was observed by Hematoxylin and Eosin (HE) and Nissl staining as previously described (Yu et al., 2023). Sections were incubated with a primary antibody against neuronal nuclei (NeuN, 1:200 dilution, Beyotime, China) at 4°C overnight, followed by PBS washing. Then, a Cy3-conjugated secondary antibody (1:500, Servicebio, China) was applied for 1 h at room temperature. Fluorescence was detected under a fluorescence microscope (Nikon, Japan).

### 2.8 Enzyme-linked immunosorbent assay (ELISA)

LDH (Beyotime, China), superoxide dismutase (SOD, Dojindo, Japan), catalase (CAT), glutathione peroxidase (GSH-Px), and hydrogen peroxide (H_2_O_2_) concentrations were measured by respective ELISA kits (Nanjing Jiancheng Biological Engineering Research Institute, China), following the provided protocols.

### 2.9 Hippocampal CAIII gene knockdown in mice

Recombinant AAV vectors encoding CAIII shRNA (5′-GGTTCACTGGAATCCAAAGTA-3′) or non-specific control shRNA (5′-CGCTGAGTACTTCGAAATGTC-3′) were synthesized by Shanghai Genechem Co., Ltd. Mice were anesthetized with 1.2% isoflurane and placed on a stereotaxic apparatus (RWD, China) with a heating pad to maintain body temperature at 37°C. A cranial incision was created by an electric drill (RWD, China), followed by the precise injection of 2 × 10^9 v.g. AAV into each hippocampus (AP: −1.85 mm, ML: ±1.60 mm, DV: −1.90 mm) at 0.08 μL/min via a glass micropipette. The syringe (Gaoge, China) remained for 10 min before being slowly withdrawn. After bilateral injections, the incision was sutured, and the mice were kept warm until recovery.

### 2.10 Cell culture and treatment

PC12 cells, obtained from Shanghai Fuheng Biotechnology, were authenticated before use through standard cell line identification methods. The CAIII knockdown (CAIII sh), CAIII overexpressing (CAIII OE), and negative control (NC) PC12 cells were transfected with lentiviral vectors. Cells were cultured in RPMI-1640 medium supplemented with 10% fetal bovine serum, 100 μg/mL penicillin, 100 μg/mL streptomycin, and 1 μg/mL puromycin, and maintained in a humidified 5% CO_2_ incubator at 37°C. Previous studies indicated that PC12 cell growth was inhibited by elevated glucose and palmitic acid levels. A combination of 100 mM glucose and 200 μM palmitic acid (G100F200) was identified as the threshold for significant growth inhibition, establishing an *in vitro* diabetes model. The CAIII sh, CAIII OE, and NC cells were exposed to four conditions for 24 h: normal culture medium (Con group), high-glucose/high-lipid medium (G100F200 group), solvent control (DMSO group), and 60 μM SP (SP group).

### 2.11 RNA sequencing (RNA-seq)

Total RNA was extracted from PC12 cells using the RNA Extraction Kit (Takara, Japan) according to the manufacturer’s protocol. RNA quantity and integrity were evaluated with a NanoDrop ND-2000/Qubit 2.0, and Agilent 4,200 TapeStation (Agilent Technologies, USA). Only high-quality samples were used for transcriptome sequencing. Double-stranded cDNA libraries were constructed using the TruSeq® RNA Sample Preparation Kit (Illumina, USA), following the recommended protocol. In brief, mRNA containing polyadenylated tails was enriched using magnetic beads conjugated with poly-T oligonucleotides. The resulting libraries were quantified with a Qubit® 2.0 Fluorometer (Life Technologies, USA), and library quality—including fragment size and molar concentration—was assessed using the Agilent 2,100 Bioanalyzer (Agilent Technologies, USA). Sequencing clusters were generated on a cBot system after diluting the libraries to 10 pM, and paired-end sequencing was performed on the Illumina HiSeq X Ten platform (Illumina, USA). All steps related to library preparation and sequencing were conducted at Shanghai Biotechnology Corporation. Raw sequencing reads were initially subjected to quality control, during which rRNA sequences, adapter contaminants, short fragments, and low-quality reads were removed. Clean reads were then aligned to the human reference genome (GRCh38) using HISAT2 (version 2.0.4) (Kim et al., 2015), allowing up to two mismatches. Transcript assembly and quantification were performed with StringTie (version 1.3.0) (Pertea et al., 2015; Pertea et al., 2016) using the corresponding gene annotation to calculate FPKM (Fragments Per Kilobase of transcript per Million mapped reads) values for annotated genes. Differential gene expression was assessed using edgeR (Robinson et al., 2010), followed by functional annotation using Kyoto Encyclopedia of Genes and Genomes (KEGG) pathway and Gene Ontology (GO) enrichment analyses. The significance threshold for multiple comparisons was controlled using the false discovery rate (FDR) method. Fold change values were calculated based on FPKM levels in individual samples. Genes were considered differentially expressed if they met both criteria: FDR ≤ 0.05 and fold change≥2. Data processing was conducted using R software (version 4.3.1).

### 2.12 Cell viability assay

Cell viability was measured by CCK-8 assay kit (Dojindo, Japan). The CAIII sh, CAIII OE, and NC PC12 cells were plated at 4000 cells/well in a 96-well plate and cultured for 24 h. They were then treated with normal culture medium (Con group), high-glucose and high-fat culture medium (100 mM glucose +200 μM palmitic acid, G100F200 group), solvent control (DMSO group), and 60 μM SP (SP group) for 24 h. Following treatment, 10 μL of CCK8 reagent was added to each well and incubated for 2 h in the dark. The absorption was determined at 450 nm using a microplate reader (Biotek, USA).

### 2.13 Flow cytometry

Apoptosis was analyzed by flow cytometry using dual staining with Annexin V-FITC (to detect early apoptotic cells) and propidium iodide (PI) (to identify late apoptotic and necrotic cells) (Thermo Scientific, USA). The quadrants were interpreted as follows: Q1 (upper left): PI^+^/Annexin V^−^ (necrotic cells), Q2 (upper right): PI^+^/Annexin V^+^ (late apoptotic cells), Q3 (lower right): PI^−^/Annexin V^+^ (early apoptotic cells), and Q4 (lower left): PI^−^/Annexin V^−^ (viable cells).

### 2.14 Measurement of mitochondrial membrane potential and mitochondrial ROS

Mitochondrial membrane potential was assessed by TMRE (tetramethylrhodamine ethyl ester) staining in PC12 cells seeded at 5000 cells/well on 12-well plates with coverslips and incubated for 24 h. Following treatment with normal culture medium, G100F200, DMSO, or SP, cells were incubated with TMRE working solution (Beyotime, China) for 10 min. Cells were then stained with 10 μg/mL Hoechst 33258 (Yeasen, China) for 10 min. Fluorescent images were captured using a fluorescence microscope (Nikon, Japan). Mitochondrial ROS levels were measured using MitoSOX Red (Thermo Scientific, USA). After treatment with the respective media, cells were incubated with 5 μM MitoSOX for 10 min, followed by 0.1 μM Mito-Tracker Green (Beyotime, China) for 30 min and 10 μg/mL Hoechst 33258 for 10 min. After washing with HBSS, fluorescence was analyzed under a fluorescence microscope (Nikon, Japan).

### 2.15 RNA extraction and real-time PCR

Total RNA from the brain and the cells were extracted using an RNA Extraction Kit (Takara, Japan) followed by the manufacturer’s instructions. The cDNA was synthesized from 1,000 ng RNA using the 1st Strand cDNA Synthesis SuperMix (Yeasen, China). mRNA levels were quantified via SYBR Green (Yeasen, China) real-time PCR and normalized to *β*-actin using the 2^−∆∆Ct^ method. Primer sequences: mouse CAIII (F 5′-GAATCTCAGCACTCCTACTTTCA-3′; R 5′-GTCCGCATACTCCTCCATAC-3′), mouse β-actin (F 5′-CCTCTATGCCAACACAGT-3′; R 5′-AGCCACCAATCCACACAG-3′), rat CAIII (F 5′-TGCCGGGACTATTGGACCTA-3′; R 5′-ATTCTCTGCACTGGCGAACA-3′), rat β-actin (F 5′-CCTCTATGCCAACACAGT-3′; R 5′-AGCCACCAATCCACACAG-3′).

### 2.16 Western blotting

Proteins from the brains and the cells were extracted using radioimmunoprecipitation assay (RIPA) buffer as previously described (McCrimmon et al., 2012). Protein concentrations were determined by BCA method (Thermo Scientific, USA). Proteins were separated by sodium dodecyl sulphate polyacrylamide gel electrophoresis (SDS-PAGE), transferred to polyvinylidene fluoride (PVDF) membranes (Millipore, USA), and probed with anti-CAIII (1:500; Santa Cruz, USA), anti-p-AMPK (1:1000; Cell Signaling, USA), anti-AMPK (1:1000; Cell Signaling, USA), anti-Sirt1 (1:1000; Abcam, UK), anti-PGC-1α (1:1000; Cell Signaling, USA), anti-Bcl-2 (1:1000; Cell Signaling, USA), anti-Bax (1:1000; Cell Signaling, USA), cleaved-Caspase 3 (1:1000; Cell Signaling, USA), Bcl-xl (1:1000; Cell Signaling, USA), and anti-*β*-actin (1:2000; Cell Signaling, USA) antibodies. ECL (Bio-Rad, USA) was used for imaging, and the protein bands were analyzed using Image J software.

### 2.17 Statistical analysis

Data were described as mean ± standard error of mean (SEM). Statistical comparisons were made using Student’s t-test for two-group analysis and one-way ANOVA for multiple groups. Analysis and graphing were conducted with GraphPad Prism 9.0, EthoVision, Image J, and R software. *p* < 0.05 was considered statistically significant.

## 3 Results

### 3.1 SP attenuated hippocampal neuronal damage in diabetic mice

The structure of 9-PAHSA and S-9-PAHSA (SP) are depicted in Figure 1A, and the experimental timeline is shown in Figure 1B. The hippocampus is an essential region for memory and the loss of hippocampal neurons is the main pathological feature of cognitive impairment. HE staining revealed that the ND group displayed clearly, normally arranged neurons with evenly distributed basophilic granules (Nissl bodies) (Figures 1C,D). Conversely, HFD-fed mice exhibited disorganized neurons in the CA1 and CA3 regions, with irregular morphology, reduced basophilic granules, highly eosinophilic cytoplasm and dense black basophilic nuclei, indicative of neuronal damage. SP treatment ameliorated this injury in diabetic mice, as evidenced by improved cellular organization (Figures 1C–G). Nissl staining confirmed that the significant Nissl body reduction in the HFD group was significantly reduced, suggesting neurodegeneration, which was attenuated by SP (Figures 1D–G). The results implied that SP might rescue the HFD-induced hippocampal neuronal damage.

### 3.2 SP ameliorated working memory in T2DM mice through Bcl-2/Bax and AMPK/Sirt1/PGC1α pathway

**Figure 2:**
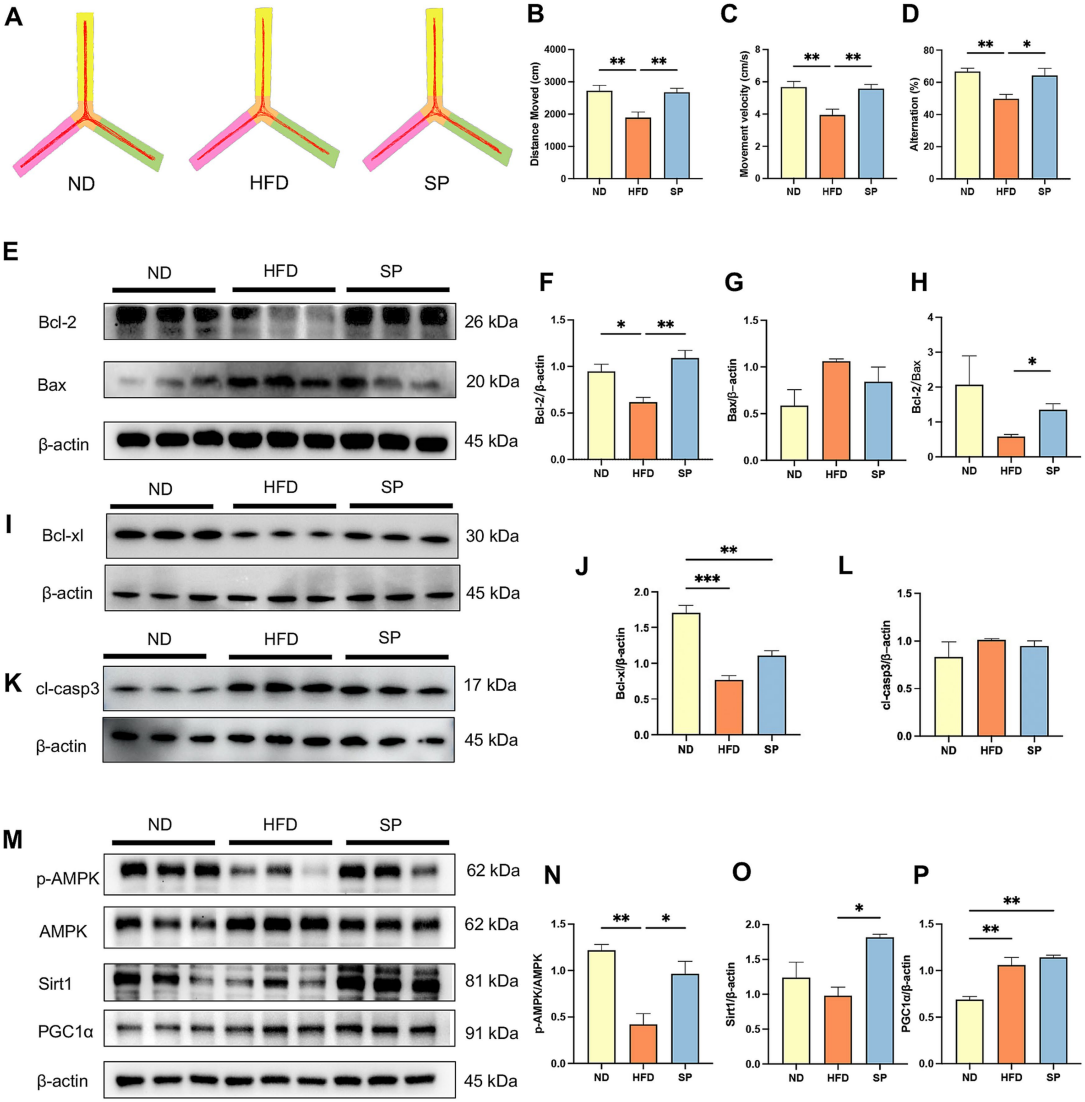
SP ameliorates cognitive disorders of T2DM mice by inhibiting apoptosis through the AMPK/Sirt1/PGC1α pathway. **(A)** Representative Y-maze track images from mice, *n* = 8; The total distance moved **(B)**, mean velocity **(C)**, and alternation **(D)** of the mice in three groups in the Y maze test. **(E–L)** Western blot analysis of Bcl-2 and Bax, Bcl-xl, and cleaved-Caspase 3 (cl-casp3) expression in mouse hippocampus, *n* = 3; **(M–P)** Western blot analysis of p-AMPK, AMPK, Sirt1, and PGC1α expression, *n* = 3. Data are presented as mean ± SEM. *p < 0.05, **p < 0.01, ***p < 0.001, ****p < 0.0001.

Y maze test was used to assess working memory, and representative trajectories are shown in Figure 2A. HFD-fed mice showed reduced total distance, movement velocity, and alternation percentage compared to ND-fed mice, while SP treatment reversed these deficits (Figures 2B–D, all *p* < 0.05). This indicated that SP enhanced working memory in diabetic mice.

Next, we found that SP increased Bcl-2 (Figures 2E,F, *p* < 0.01) and Bcl-xl (Figures 2I,J, *p* > 0.05) expression, decreased Bax expression (Figures 2G, *p* > 0.05) and cleaved-Caspase 3 (cl-casp3) (Figures 2K,L, *p* > 0.05), and remarkably elevated the Bcl-2/Bax ratio (Figure 2H, *p* < 0.05) in the hippocampus, attenuating neuronal apoptosis in the hippocampus.

In addition, AMPK phosphorylation was significantly diminished in diabetic mice (Figures 2M–P, *p* < 0.01). SP intervention rescued the inhibition of AMPK phosphorylation in the HFD group (Figures 2M,N, *p* < 0.05), significantly increased downstream Sirt1 expression (Figure 2O, *p* < 0.05), and slightly elevated PGC1α expression (Figure 2P, *p* > 0.05).

### 3.3 Effects of SP on serum and hippocampal oxidative stress

**Figure 3:**
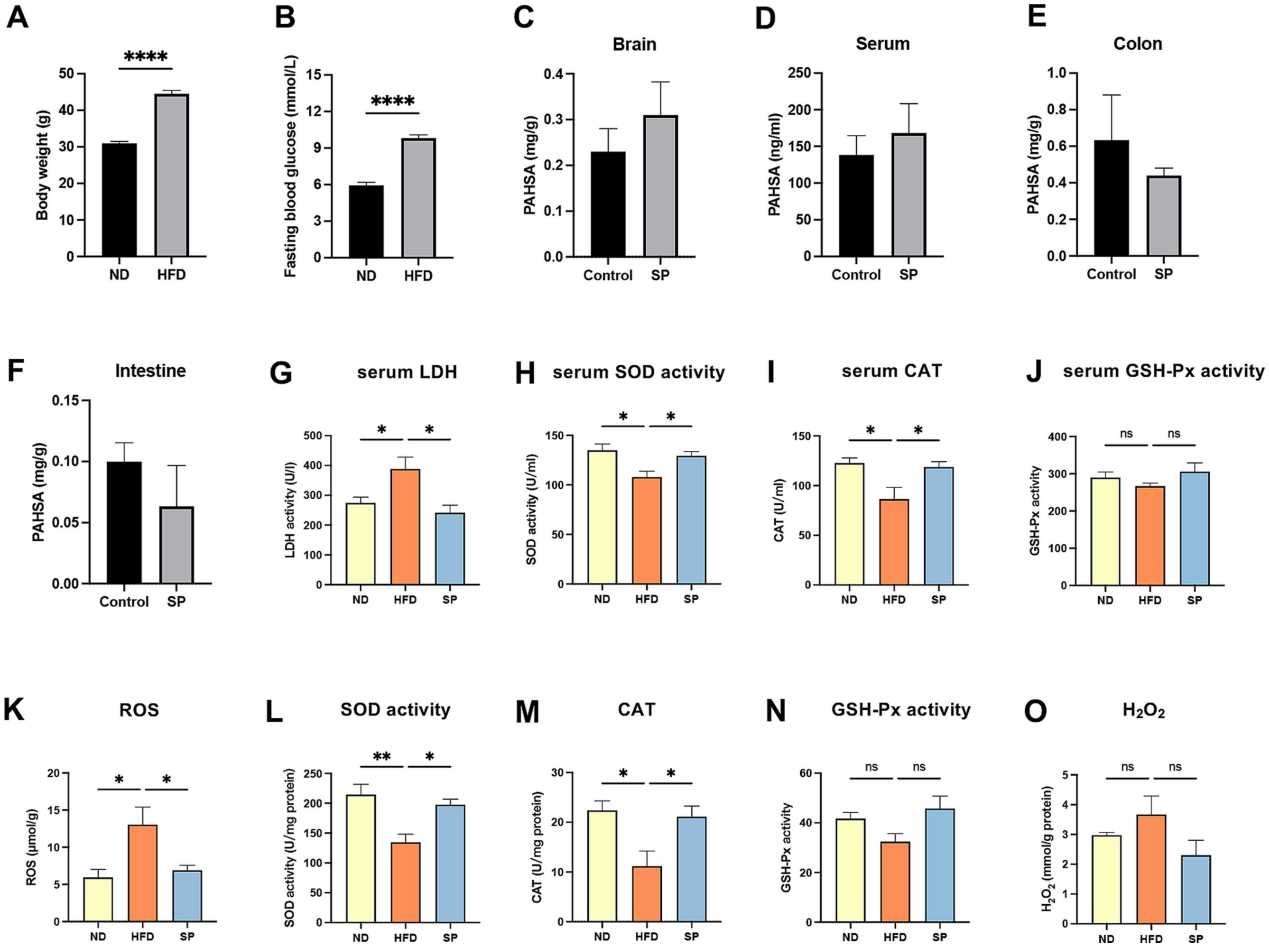
SP improves oxidative stress in T2DM mice. Body weight **(A)** and fasting glucose **(B)** in mice on normal (*n* = 15) or HFD (*n* = 30) for 5 months; Tissue SP levels in brain **(C)**, serum **(D)**, colon **(E)**, and intestine **(F)**, detected by UPLC-MS/MS after the administration of vehicle or SP (30 mg/kg/d), *n* = 3; Serum LDH activity **(G)**, SOD activity **(H)**, CAT **(I)**, and GSH-Px activity **(J)** in ND, HFD, and SP groups, *n* = 5; Hippocampal ROS **(K)**, SOD activity **(L)**, CAT **(M)**, GSH-Px **(N)** and H_2_O_2_ **(O)** levels, *n* = 3 ~ 4; Data are presented as mean ± SEM. *p < 0.05, **p < 0.01, ***p < 0.001, ****p < 0.0001.

Mice on a high-fat diet showed significantly increased body weights (44.51 ± 5.06 g vs. 30.97 ± 1.19 g) and fasting blood glucose levels (9.82 ± 1.39 mmoL/L vs. 5.95 ± 0.98 mmoL/L) compared to those on a normal diet (Figures 3A,B, all *p* < 0.0001). Of the 30 HFD-fed mice, 28 had fasting glucose levels exceeding 8.0 mmoL/L, resulting in a 93.3% success rate for diabetes modeling. These findings confirm that chronic HFD feeding substantially elevated both body weight and blood glucose. In HFD-fed mice treated with SP, serum and brain SP levels were increased by 34.8 and 21.7%, respectively, while levels in the small intestine and colon were reduced by 36.7 and 83.6%, respectively (Figures 3C–F, *p* > 0.05). The results suggested that SP is absorbed through the intestine and can reach the brain. Lactate dehydrogenase (LDH), a key marker of tissue damage, showed significantly increased activity in the serum of HFD mice compared to ND controls, which was attenuated by SP treatment (Figure 3G). Additionally, hippocampal reactive oxygen species (ROS) levels were lower in SP-treated mice compared to the HFD group (Figure 3K). In both serum and hippocampus, antioxidant enzyme activities of SOD and CAT were reduced in the HFD group but were elevated by SP treatment (Figures 3H,I,L,M). SP also tended to increase GSH-Px activity (Figures 3J,N), although it had no effect on H_2_O_2_ (Figure 3O). These results suggest that SP may alleviate oxidative stress and exert protective effects.

### 3.4 CAIII knockdown had no significant effects on body weight and fasting blood glucose in diabetic mice

**Figure 4:**
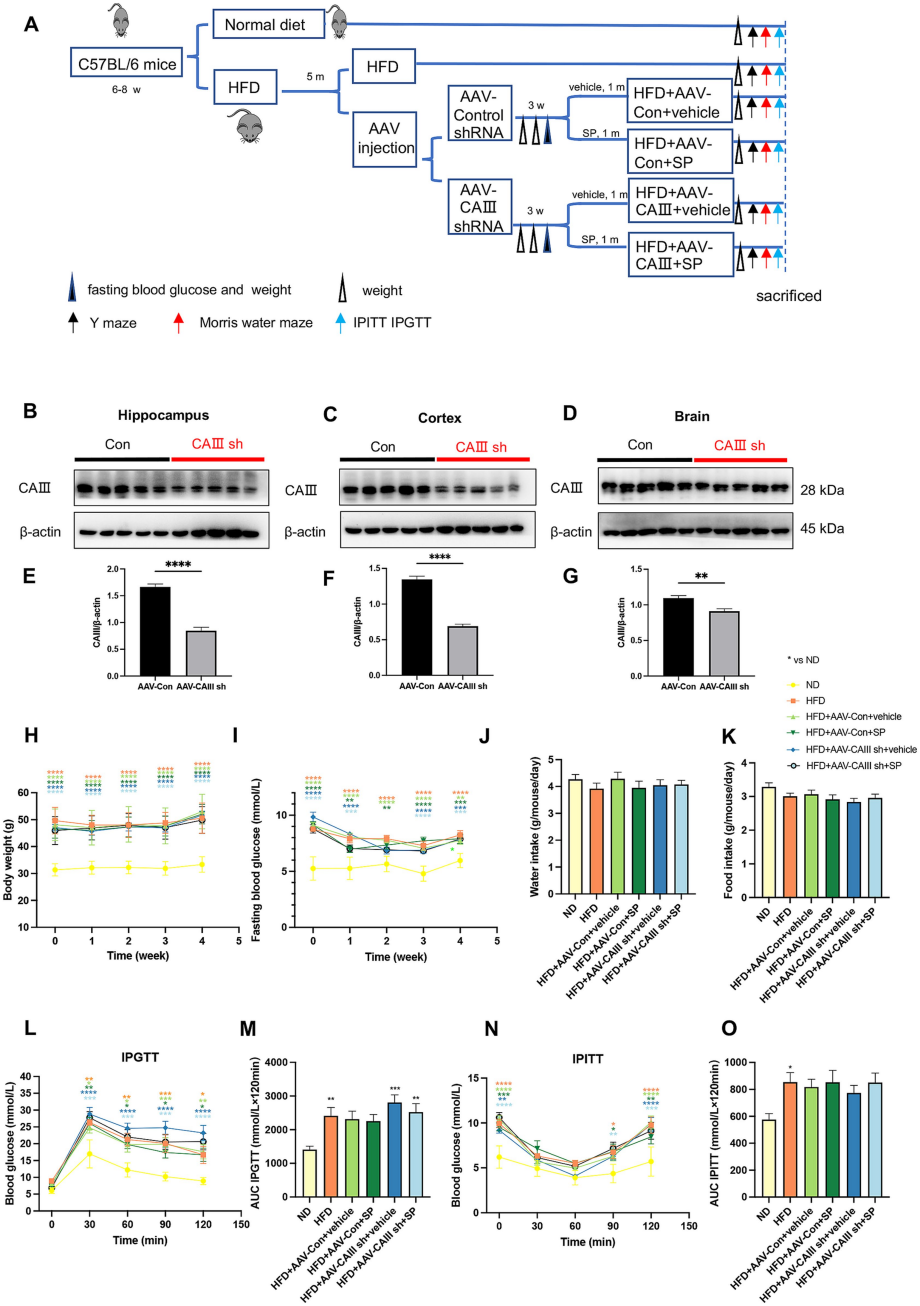
The knockdown of CAIII in the hippocampus and the effects of SP during the administration. **(A)** Overview of the experimental design; **(B–G)** protein expression and statistical analysis of CAIII in the hippocampus **(B,E)**, cortex **(C,F)** and the remaining brain (excluding the hippocampus and cortex) **(D,G)** of mice following injection with control or CAIII shRNA, *n* = 5; Body weight **(H)**, fasting glucose **(I)**, water intake **(J)**, and food intake **(K)** in mice during SP treatment, *n* = 15; **(L,M)** IPGTT results and AUC, *n* = 9; **(N,O)** IPITT results and AUC, *n* = 9. Data are presented as mean ± SEM. *p < 0.05, **p < 0.01, ***p < 0.001, ****p < 0.0001.

We observed a reduction in CAIII expression in the hippocampus of HFD-fed mice, whereas treatment with SP effectively mitigated the HFD-induced downregulation of CAIII (Supplementary Figures S2A,B, *p* < 0.01), suggesting that CAIII may be involved in the protective effects of SP. To further investigate this possibility, we performed bilateral hippocampal knockdown of CAIII in T2DM mice following the protocol (Figure 4A). HFD mice were bilaterally injected with AAV-Con sh or AAV-CAIII shRNA. After 3 weeks, mRNA and protein expression of CAIII in the hippocampus, cortex, and remaining brain regions (excluding the hippocampus and cortex) were significantly reduced in the AAV-CAIII sh group compared to the AAV-Con sh group (Figures 4B–G; Supplementary Figures S3A–C). These findings confirm the effective knockdown of CAIII in these brain areas following AAV-CAIII shRNA injection. AAV injection had no observable effect on weekly body weight or fasting blood glucose levels in any group (Supplementary Figures S3D,E). Weekly body weight and fasting blood glucose of HFD-fed mice were significantly higher compared to ND-fed mice, with no significant variations observed within the HFD group (Figures 4H,I). Daily water intake and food intake showed no difference across all groups (Figures 4J,K, *p* > 0.05). In IPGTT, blood glucose peaked 30 min post-injection, with HFD-fed mice showing higher levels than ND-fed mice at all time points, but no AUC differences among HFD-fed groups (Figures 4L,M). IPITT revealed no significant differences in insulin sensitivity among HFD-fed groups (Figures 4N,O). These results indicated that SP had no significant effect on glucose tolerance and insulin sensitivity.

### 3.5 CAIII knockdown abolished the improvement of SP on diabetic mice cognition

**Figure 5:**
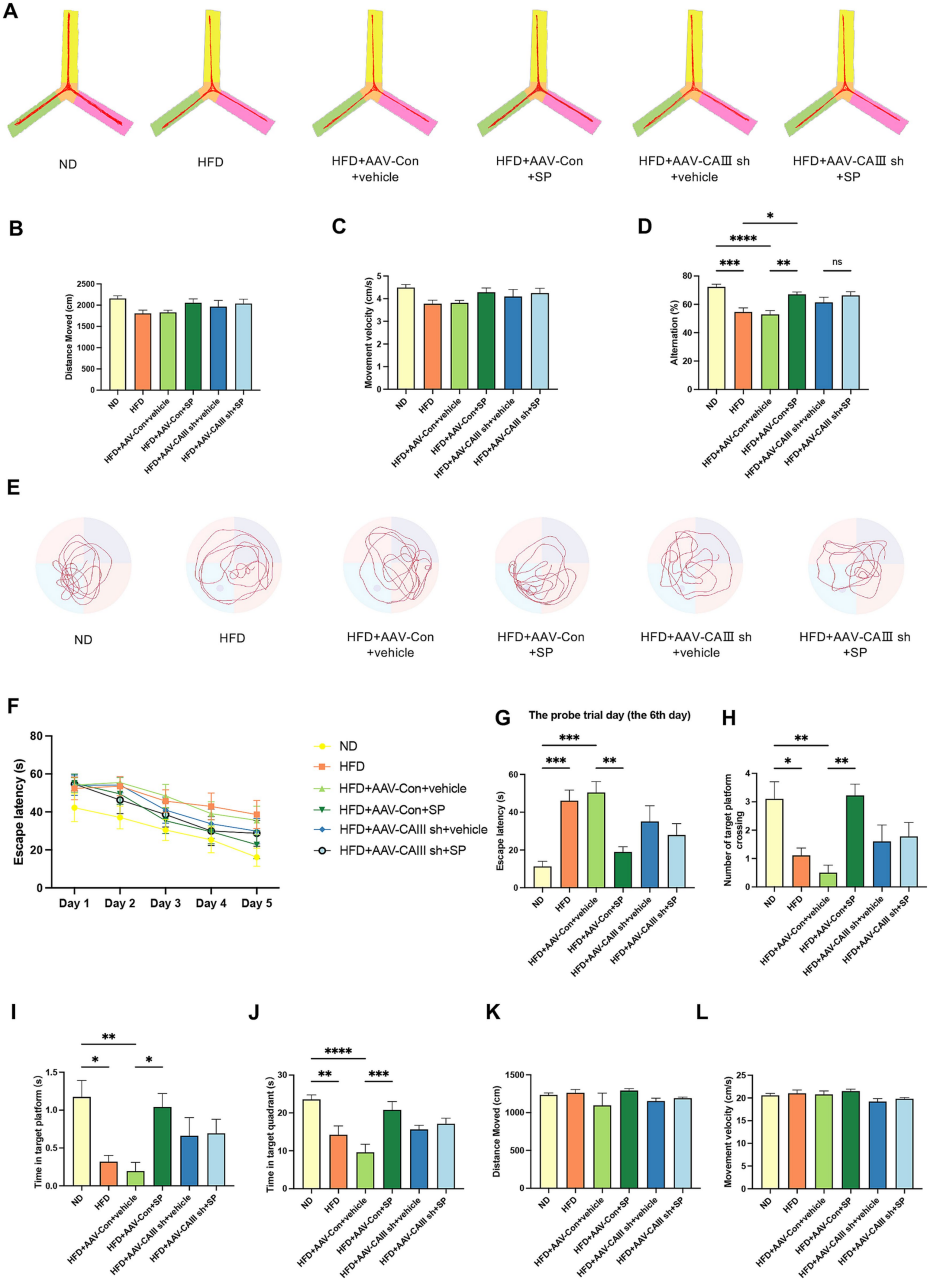
CAIII-mediated the improvement of SP in the cognitive impairment of T2DM mice. **(A)** Y-maze test track images from mice, *n* = 10 ~ 11; The total distance moved **(B)**, velocity **(C)**, and alternation **(D)** for different groups in the Y maze test; **(E)** Representative tracks of each group on the probe trial day in the Morris water maze test; **(F)** latency to platform over 5 training days; Escape latency **(G)**, platform crossings **(H)**, time at target platform **(I)**, time in target quadrant **(J)**, total distance **(K)** and movement velocity **(L)** of each group on the probe trial day, *n* = 8 ~ 10; Data are presented as mean ± SEM. *p < 0.05, **p < 0.01, ***p < 0.001, ****p < 0.0001.

Y-maze test showed that compared to the ND group, the total distance (*p* > 0.05), movement velocity (p > 0.05), and alternation percentage (*p* < 0.05) decreased in the HFD group and HFD + AAV-Con+vehicle group (Figures 5A–D). SP administration improved cognitive performance (Figure 5D, *p* < 0.01), except for no change in alternation percentage between HFD + AAV-CAIII+vehicle and HFD + AAV-CAIII+SP groups. It suggested that SP ameliorates spatial memory in diabetic mice, with CAIII knockdown in the hippocampus negating this benefit.

The trajectories of MWM test are shown in Figure 5E. HFD-fed mice demonstrated increased latency compared to ND controls across training days (Figure 5F). On the probe trial day, the escape latency elevated in the HFD and the HFD + AAV-Con+vehicle group, which was remarkably reduced by SP treatment, but this effect was abolished with CAIII knockdown (Figure 5G, *p* < 0.05). HFD group and the HFD + AAV-Con+vehicle group also showed significant fewer platform crossings and decreased time in the platform and the target quadrant versus the ND group, improvements that were observed with SP administration (Figures 5H–J, *p* < 0.05). No obvious difference in total distance and velocity were noted among groups (Figure 5K,L, *p* > 0.05). These findings further confirmed that CAIII may mediate the protective effect of SP on cognitive function of diabetic mice.

### 3.6 SP reduced ROS and ameliorated neuronal damage in mouse hippocampus via CAIII-mediated Bcl-2/Bax and AMPK/Sirt1/PGC1α

**Figure 6:**
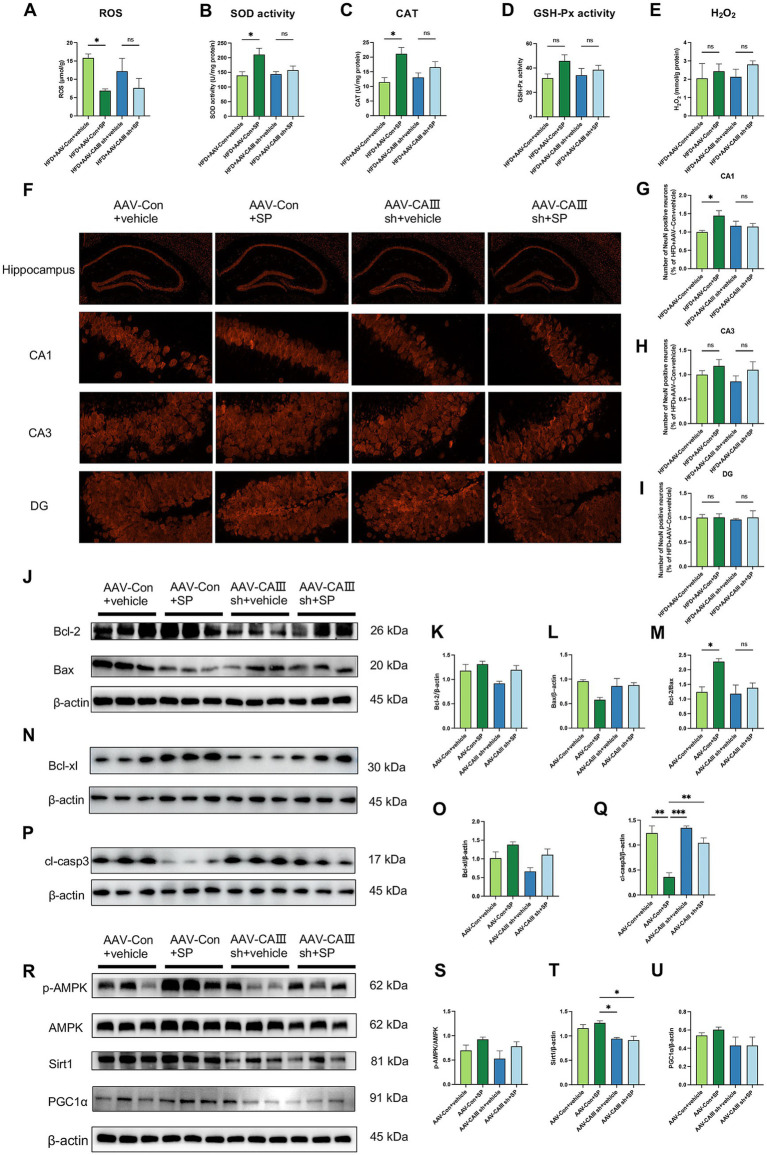
SP ameliorated neuronal injury and apoptosis via CAIII-mediated Bcl-2/Bax and AMPK/Sirt1/PGC1α pathway. Hippocampal ROS **(A)**, SOD activity **(B)**, CAT **(C),** and GSH-Px **(D)** and H_2_O_2_ **(E)** in mice, *n* = 4 ~ 5. **(F)** NeuN immunostaining in hippocampal sections across groups; NeuN-positive neuron percentages in CA1 **(G)**, CA3 **(H)**, and DG **(I)** regions, normalized to the HFD + AAV-Con+vehicle group, *n* = 4; **(J–Q)** Western blot analysis of Bcl-2, Bax, Bcl-xl, and cleaved-Caspase 3 (cl-casp3) in mouse hippocampus, *n* = 3; **(R-U)** Western blot analysis of *p*-AMPK, AMPK, Sirt1 and PGC1α, *n* = 3. Data are presented as mean ± SEM. *p < 0.05, **p < 0.01, ***p < 0.001, ****p < 0.0001.

Hippocampal ROS levels were significantly elevated in the HFD and HFD + AAV-Con+vehicle groups but were notably reduced with SP treatment. This SP-induced reduction in ROS was attenuated in CAIII knockdown mice (Figure 6A). SP also enhanced SOD activity and CAT, effects not seen in CAIII knockdown mice (Figures 6B,C). A similar trend was also found in GSH-Px (Figure 6D, *p* > 0.05), while SP had no significant effect on H_2_O_2_ (Figure 6E, *p* > 0.05). These findings indicate that SP’s neuroprotection may involve CAIII-mediated modulation of oxidative stress. NeuN staining revealed disorganized neurons in the CA1 and CA3 regions of the HFD + AAV-Con+vehicle group, which were more orderly in the HFD + AAV-Con+SP group. This beneficial effect of SP was not observed in CAIII knockdown mice (Figure 6F). Additionally, the proportion of NeuN-positive cells was higher in both the CA1 (Figure 6G, *p* < 0.05) and CA3 regions (Figure 6H, *p* > 0.05) in the HFD + AAV-Con+SP group compared to the HFD + AAV-Con+vehicle group, with CAIII knockdown reducing this protective effect (Figures 6G–I, *p* > 0.05). Furthermore, the SP-induced increases in Bcl-2/Bax ratio and Bcl-xl were diminished in CAIII knockdown mice (Figures 6J–O), while the cleaved-Capase 3 decreased by SP was increased after CAIII knockdown (Figures 6P,Q, *p* > 0.05). Likewise, the inhibitory impact of SP on the AMPK pathway in diabetic mice was also decreased (Figures 6R–U, *p* > 0.05). These results suggested that CAIII played a crucial role in mediating the neuroprotective and anti-apoptotic effects of SP.

### 3.7 CAIII mediated SP protection against cellular damage and mitochondrial dysfunction in PC12 cells

**Figure 7:**
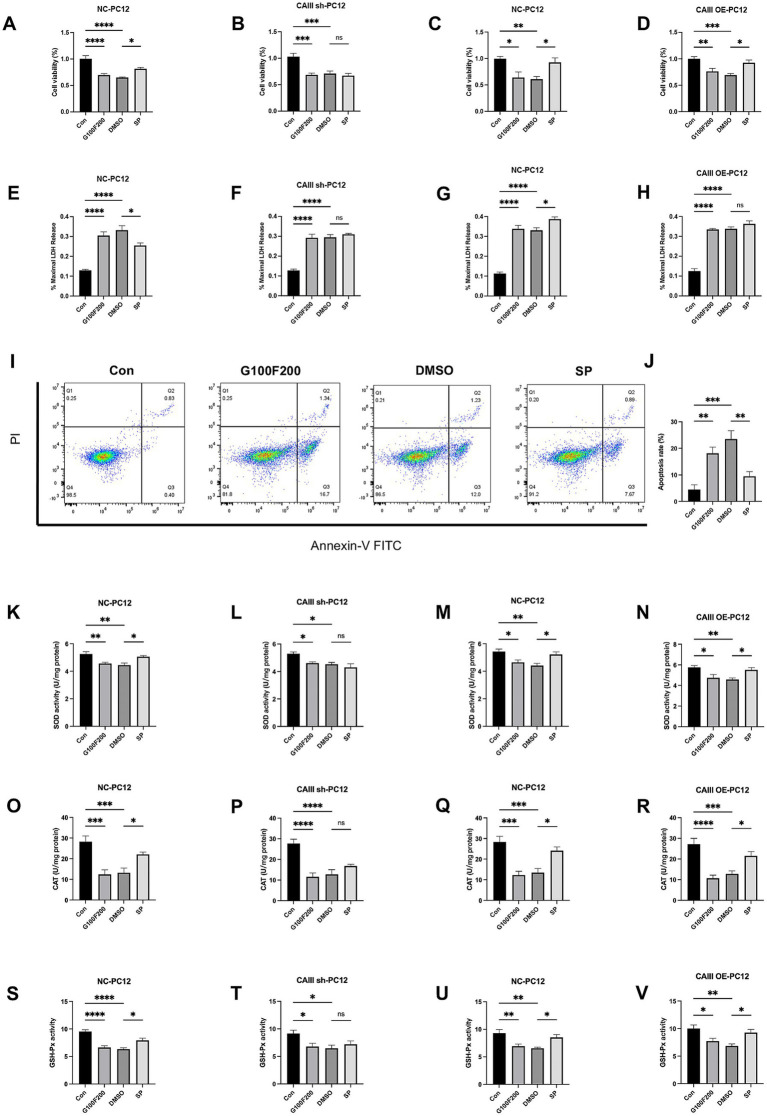
The effects and mechanism of SP on cell viability, LDH release, and mitochondrial production in PC12 cells. NC, CAIII sh, and CAIII OE cells were exposed to standard medium (Con), high-glucose and high-fat medium (100 mM glucose + 200 μM palmitic acid, G100F200), G100F200 with DMSO, or G100F200 with 60 μM SP for 24 h. **(A–D)** Cell viability of CAIII sh cells, CAIII OE cells, and NC cells, *n* = 6; **(E–H)** LDH release of CAIII sh cells, CAIII OE cells and NC cells, *n* = 5 ~ 6; **(I)** Flow cytometric analysis of apoptosis using Annexin V-FITC/PI staining. **(J)** Quantification of apoptosis rate based on flow cytometry results. SOD activity **(K–N)**, CAT **(O–R)**, and GSH-Px **(S–V)** of CAIII sh cells, CAIII OE cells, and NC cells, *n* = 6. Data are presented as mean ± SEM. *p < 0.05, **p < 0.01, ***p < 0.001, ****p < 0.0001.

Consistent with the *in vivo* findings, our *in vitro* experiments revealed that a diabetes-mimicking environment led to a reduction in CAIII expression, which was alleviated by SP treatment (Supplementary Figures S2C,D, *p* < 0.05). Lentiviral transfection successfully generated CAIII knockdown (CAIII sh), overexpressing (CAIII OE), and their negative control (NC) PC12 cells, confirmed at both the mRNA and protein levels (Supplementary Figures S4A–F, *p* < 0.05). Cell viability was reduced in the G100F200 and DMSO groups but increased in NC and CAIII OE cells (Figures 7A,C,D, *p* < 0.05). SP did not significantly affect cell viability in CAIII sh cells under diabetic conditions (Figures 7B, *p* > 0.05). Elevated LDH release was observed under high glucose and lipid conditions in NC cells (Figures 7E–H, *p* < 0.001). SP intervention significantly lowered LDH release in NC cells exposed to diabetic conditions (Figure 7E, *p* < 0.05), but this inhibitory effect was negated by CAIII knockdown (Figure 7F, *p* > 0.05). Additionally, there was a tendency for SP to increase LDH in CAIII OE cells (Figure 7H, *p* > 0.05). Flow cytometric analysis further demonstrated that SP significantly attenuated apoptosis induced by high-fat and high-glucose conditions (Figures 7I,J, *p* < 0.01). In NC cells, antioxidant enzymes including SOD, CAT, and GSH-Px were significantly reduced in G100F200 and DMSO group compared to controls (Figures 7K–U, *p* < 0.05). SP treatment notably reversed this decline (Figures 7K,O,S, *p* < 0.05). However, in CAIII knockdown cells, SP showed no significant effect (Figures 7L,P,T, *p* > 0.05). In contrast, in CAIII OE cells, SP continued to restore antioxidant enzyme levels (Figures 7N,R,V, *p* < 0.05). Therefore, CAIII might mediate the protective effects on neuronal viability and cellular damage mitigation by SP in vitro.

**Figure 8:**
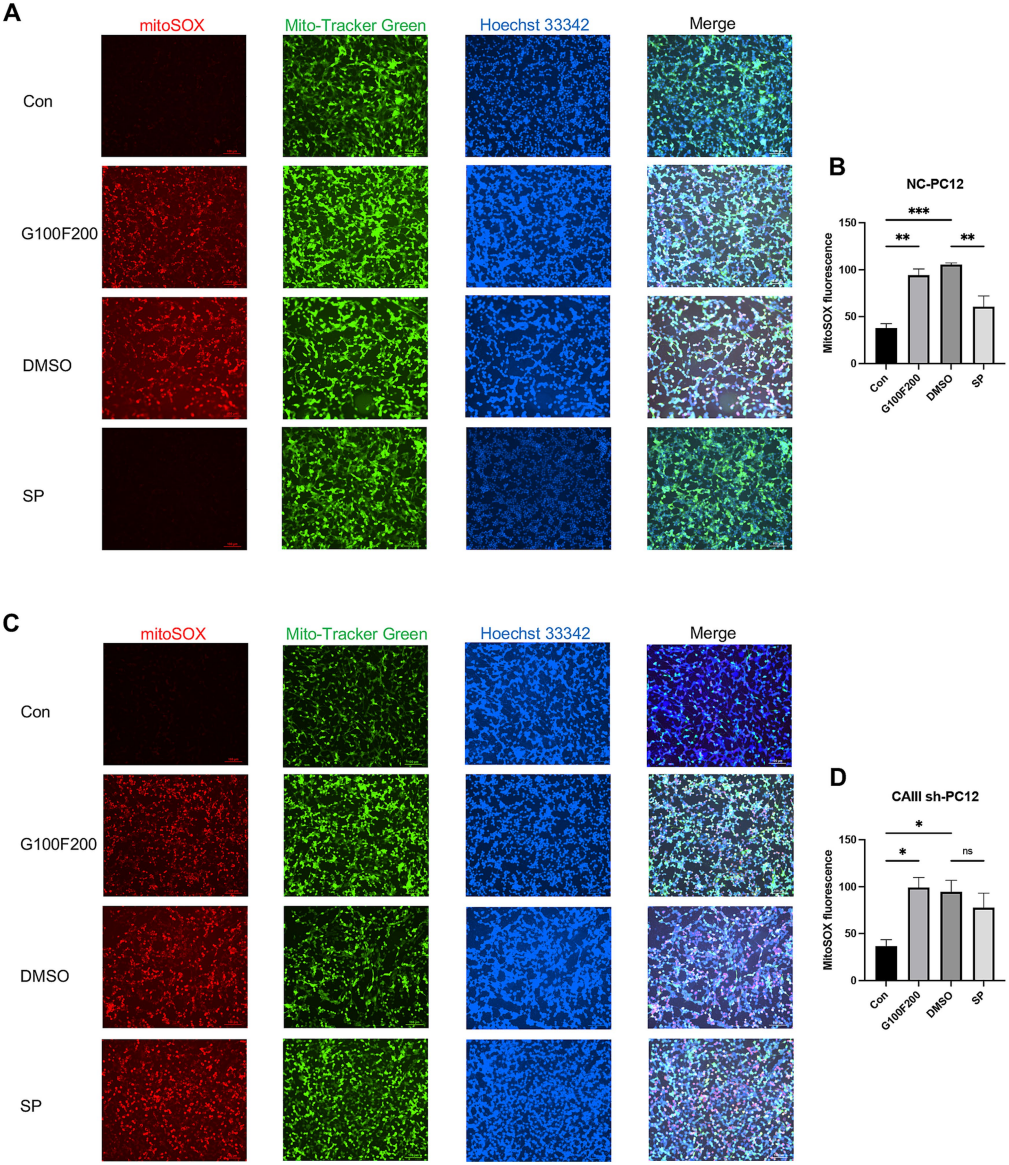
The effects and mechanism of SP on mitochondrial production in PC12 cells. MitoSOX Red immunofluorescence for mitochondrial ROS and quantification in NC **(A,B)** and CAIII sh **(C,D)** cells, *n* = 3. Data are presented as mean ± SEM. *p < 0.05, **p < 0.01, ***p < 0.001, ****p < 0.0001.

Our in vivo experiments indicated that CAIII influences hippocampal ROS production. Given mitochondria’s central role in ROS generation, we detected ROS levels in mitochondria. Mitochondrial ROS were markedly elevated in NC cells under high-glucose and high-fat stimulation but were attenuated by SP (Figures 8A,B, *p* < 0.05). However, SP had no significant effect on mitochondrial ROS in CAIII knockdown cells (Figures 8C,D, *p* > 0.05). Subsequently, we assessed the mitochondrial membrane potential by TMRE staining, which revealed high intensity in controls and diminished intensity under diabetic conditions. SP treatment restored the loss of mitochondrial membrane potential in the diabetic environment (Supplementary Figures S5A,B, *p* < 0.0001). This beneficial effect of SP was absent in CAIII knockdown cells (Supplementary Figures S5C,D, *p* > 0.05). These results suggest that CAIII is essential for the impact of SP on mitochondrial function and ROS reduction in diabetic conditions.

### 3.8 CAIII modulated SP-induced gene expression and pathway activation in PC12 cells

Volcano plots and heatmaps were used to visualize differentially expressed genes (DEGs) between DMSO- and SP-treated groups. Thirteen genes were significantly altered in NC cells following SP treatment, including five upregulated and eight downregulated transcripts (Supplementary Figures S6A–D, FDR-adjusted *p* < 0.05). The most prominently upregulated genes included *Pfkfb3*, *Mrfap1*, *RT1-M6-1*, *1810024B03Rik*, and *Fbxo41*. Among these, Pfkfb3 has been implicated in multiple biological processes, particularly in the regulation of the AMPK signaling pathway. In CAIII sh cells, five genes were differentially expressed in the SP group, with 3 upregulated (*Srm*, *Cebpb*, *Oca2*) and 2 downregulated (*LOC361108*, *Mgp*) (Supplementary Figures S6A–D, FDR-adjusted *p* < 0.05), involving in cellular process and various pathways. The UpSet plot further illustrates the intersections of DEGs in DMSO- or SP-treated NC cells and CAIII sh cells (Supplementary Figure S6E).

**Figure 9:**
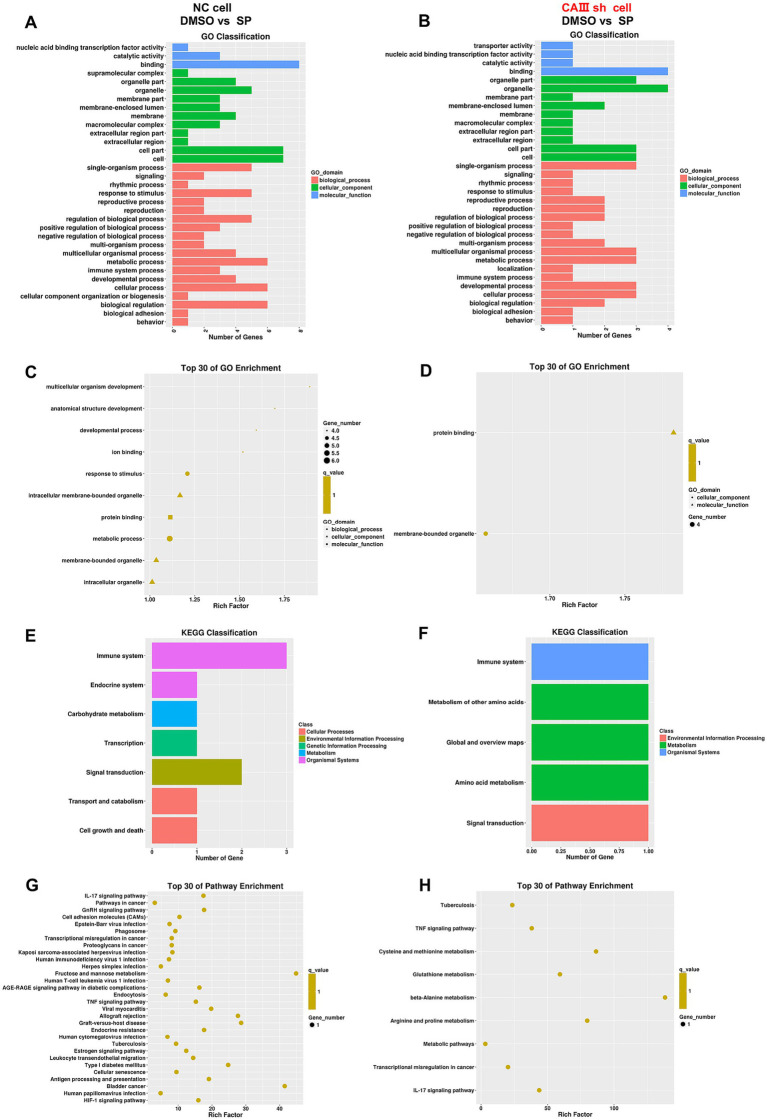
Differentially expressed genes of biological processes and pathways after SP treatment in NC and CAIII sh PC12 cells. Graphical representation of GO classification for biological processes, cellular components, and molecular functions **(A,B)** and GO enrichment analysis **(C,D)** for differential gene expression between DMSO and 60 μM SP groups in NC and CAIII sh cells, *n* = 3; KEGG pathway classification **(E,F)** and KEGG enrichment scatterplots **(G,H)** for the same comparison, *n* = 3. Data are presented as mean ± SEM. *p < 0.05, **p < 0.01, ***p < 0.001, ****p < 0.0001.

GO analysis revealed that in NC cells, SP and DMSO treatments induced differential gene expression across various biological processes, cellular components, and molecular functions (Figure 9A). In contrast, in CAIII sh cells, gene expression shifted, with a decrease in genes related to signaling and metabolic processes, an increase in organelle-associated genes, and a reduction in catalytic activity genes (Figure 9B). These changes suggest that SP modulates neuronal processes via CAIII. GO enrichment analysis showed that SP in NC cells significantly impacted terms related to multicellular organism development, anatomical structure development, response to stimulus, and intracellular membrane-bounded organelles (Figure 9C). In CAIII sh cells, SP’s effects on protein binding and membrane-bounded organelles were greatly diminished (Figure 9D), indicating that CAIII mediates SP’s regulation of cellular processes in PC12 cells.

Pathway analysis revealed that SP influenced several pathways in NC cells, including immune response, endocrine signaling, carbohydrate metabolism, transcription, signal transduction, and cell growth and death (Figure 9E). However, following CAIII knockdown, the impact of SP on immune and signal transduction pathways was reduced, while effects on endocrine signaling, carbohydrate metabolism, and cell growth were less pronounced (Figure 9F). KEGG analysis further identified that SP affected pathways involved in diabetic complications, such as AGE-RAGE signaling, endocrine resistance, type 1 diabetes, and HIF-1 signaling (Figures 9G,H). CAIII knockdown reduced the number of pathways influenced by SP, reinforcing the role of CAIII in mediating SP’s effects on oxidative stress, cell proliferation, and apoptosis in PC12 cells.

## 4 Discussion

PAHSAs are a new type of hydroxyl fatty acid synthesized endogenously in humans and mammals, with tissue- and isomer-specific distribution (Yore et al., 2014). According to the position of the branch chain of PAHSA connected to the carbon atom, PASHAs encompass multiple isomers. 9-PAHSA can improve diabetic cardiomyopathy through enhanced autophagy and attenuated cardiac hypertrophy in db/db mice (Wang Y. M. et al., 2021). Exogenous supplementation of 9-PAHSA also shows promise in alleviating diabetes-related cognitive deficits (Wen et al., 2020). We’ve developed a novel synthesis route for S-9-PAHSA (SP), which enhances GSIS and glucose uptake, unlike its R-isomer (Aryal et al., 2021).

DRCD is a chronic complication characterized by deficits in memory, information processing speed, executive function, and attention (Biessels et al., 2014). Its etiology is multifactorial, involving oxidative stress, neuronal injury, inflammation, insulin resistance, synaptic dysfunction, gut microbiota, autophagy, and more (Feinkohl et al., 2015). The management of DRCD often mirrors that of cognitive impairment or dementia, with the efficacy of hypoglycemic agents remaining debatable (Patrone et al., 2014). Carbonic anhydrase (CA) modulates memory in mice, with activators enhancing and inhibitors impairing memory (Blandina et al., 2020). In the present study, SP ameliorates cognitive deficits in diabetic mice, which aligns with our previous work on 9-PAHSA’s effects on working memory (Wen et al., 2020). This suggests SP is a potential DRCD therapeutic, with CAIII emerging as a crucial mediator of SP’s cognitive effects.

The impact of PAHSA on glycemic control in mice is currently controversial. Studies have reported the beneficial effects of PAHSA in improving glucose tolerance and insulin sensitivity, with reduced levels in insulin-resistant and obese humans (Yore et al., 2014; Hammarstedt et al., 2018; Kellerer et al., 2021). Positive correlations between PAHSA levels and insulin sensitivity have been documented in humans and mice (Yore et al., 2014; Brezinova et al., 2020; Zhou et al., 2019). PAHSA has been found to prevent impaired GSIS, boost pancreatic *β*-cell proliferation and viability, and ameliorate glucose homeostasis (Syed et al., 2019). Prolonged intervention with 5-PAHSA promotes increased GSIS in mice and human islets (Bandak et al., 2018). Oral 5-PAHSA or 9-PAHSA administration improved glucose tolerance and increased insulin and glucagon-like peptide 1 (GLP-1) secretion in mice on a normal diet or a high-fat diet (Yore et al., 2014; Syed et al., 2018; Wang et al., 2018; Vijayakumar et al., 2017). The capacity of 5-PAHSA and 9-PAHSA to inhibit lipolysis by modulating circulating free fatty acids and to regulate hepatic glucose production has also been demonstrated in mice (Zhou et al., 2019).

Growing evidence shows that oxidative stress is a pivotal factor in DRCD pathogenesis (Zhang et al., 2023; Khan et al., 2021; Fukui et al., 2001), with heightened ROS levels correlating with neuronal damage and cognitive deficits in T2DM (Wang et al., 2010; Zhou et al., 2014). Oxidative stress escalation results from both augmented free radical production and compromised antioxidant defenses, such as diminished SOD and CAT activities in the diabetic brain (Kumar and Menon, 1993). Our prior work showed that 5-PAHSA could reduce ROS in PC12 cells, exerting an antioxidant effect (Wang J. T. et al., 2021). SP similarly lowered ROS in PC12 cells under a diabetic environment in our previous research (data not shown). Echoing these results, hippocampal ROS was found to be decreased in this study. Additionally, SP inhibited oxidative stress by reducing serum LDH and enhancing serum and hippocampal SOD activity and CAT. There was also an elevated trend of GSH-Px after SP treatment. Our *in vitro* experiments further demonstrated that SP effectively mitigated the decrease in SOD, CAT, and GSH-Px levels induced by high glucose and high fat conditions. 9-PAHSA has been noted to reverse mitochondrial dysfunction and enhance the survival of steatotic primary hepatocytes (Schultz Moreira et al., 2020). Research indicates a link between CA and oxidative stress, with CA inhibitors mitigating high-altitude oxidative stress (Ali et al., 2022) and preventing mitochondrial dysfunction along with caspase activation and cell death (Solesio et al., 2018). Our results proposed that CAIII mediated the neuroprotective effects of SP against oxidative stress both *in vivo* and in vitro, underscoring CAIII’s role in SP’s antioxidant and mitochondrial-protective actions.

AMPK, recognized as an “energy sensor,” is implicated in cognitive decline in obese and diabetic patients (Frisardi and Imbimbo, 2012; Frisardi et al., 2010). Our study revealed that SP rescued the decrease in AMPK phosphorylation and the downregulation of Sirt1 and PGC1α via CAIII. These findings indicate that SP may preserve mitochondrial homeostasis and ameliorate DRCD via the CAIII-dependent AMPK/Sirt1/PGC1α pathway. RNA-seq analysis further supported this mechanism by revealing upregulation of gene involved in AMPK signaling pathway. It has been reported that increased Bax expression and cysteine asparaginase 3 activity were observed in diabetic rats, associated with reduced neuronal density and poor performance in the Morris water maze (Yonguc et al., 2015; Jafari Anarkooli et al., 2014; Jafari Anarkooli et al., 2008; Li et al., 2002). Thus, apoptosis likely drives neuronal loss and the associated cognitive deficits observed in diabetic conditions. While CAIII has been shown to exert anti-apoptotic effects in cardiac and skeletal muscle cells (Li et al., 2021; Shang et al., 2012), its neuronal role remains elusive. Our findings demonstrate that SP, via CAIII, can elevate the Bcl-2/Bax ratio and Bcl-xl, and reduce terminal apoptotic marker cleaved caspase 3 (cl-casp 3) both in vivo and in vitro, indicating that SP’s anti-apoptotic effects were CAIII-dependent. Flow cytometry further validated these findings in vitro. In this study, hippocampal CAIII knockdown did not affect body weight and fasting glucose. Similarly, SP treatment did not influence body weight, fasting glucose, food intake, and water intake of the mice, aligning with earlier findings where 5-PAHSA and 9-PAHSA administration in normal mice did not alter intake or body weight (Syed et al., 2018). Furthermore, PAHSA treatment in HFD-fed mice showed no impact on body weight or fat mass (Zhou et al., 2019).

Our study has several limitations. First, the sample size for tissue SP content was small, necessitating larger cohorts for validation. SP concentration across all the groups needs to be determined, as well as the enzymatic activity of CAIII. Second, further studies are required to assess SP’s blood–brain barrier penetration both in vivo and in vitro. Additionally, the brain receptors for SP and the specific CAIII targets in SP’s mechanism of action remain to be identified, with the precise role of CAIII in the pathway yet to be determined.

## 5 Conclusion

In this study, we demonstrated that CAIII plays a critical role in mediating the cognitive improvements induced by SP in type 2 diabetes mice. SP alleviated hippocampal damage and improved spatial memory through CAIII-dependent activating the AMPK/Sirt1/PGC1α pathway, enhancing cellular viability and reducing oxidative stress. These findings suggest that CAIII will be a promising therapeutic target for diabetes-related cognitive disorders.

## Data Availability

The original contributions presented in the study are included in the article/Supplementary material, further inquiries can be directed to the corresponding authors.
