# Correction: S-9-PAHSA ameliorates cognitive decline in a type 2 diabetes mouse model by inhibiting oxidative stress and apoptosis via CAIII modulation

**DOI:** 10.3389/fnmol.2025.1688881

**Published:** 2025-09-09

**Authors:** Xin-Ru Wang, Shan-Shan Huang, Meng Wang, Jin-Hong Lin, Jian-Tao Wang, Jiao-Qi Ren, Cheng-Feng He, Wen-Jiao Xue, Yin Wang, Xue-Chun Wang, Yan-Li Zhang, Ji-Chang Xiao, Jing-Chun Guo, Hou-Guang Zhou

**Affiliations:** ^1^Department of Geriatric Neurology of Huashan Hospital, National Clinical Research Center for Aging and Medicine, Fudan University, Shanghai, China; ^2^Department of Translational Neuroscience, Jing'an District Centre Hospital of Shanghai, State Key Laboratory of Medical Neurobiology and MOE Frontiers Center for Brain Science, and Institutes of Brain Science, Fudan University, Shanghai, China; ^3^Department of Geriatric Medicine, Qilu Hospital of Shandong University, Jinan, Shandong, China; ^4^Key Laboratory of Organofluorine Chemistry, Shanghai Institute of Organic Chemistry, University of Chinese Academy of Sciences, Chinese Academy of Sciences, Shanghai, China

**Keywords:** S-9-PAHSA, diabetes-associated cognitive disorder, oxidative stress, mitochondrial dysfunction, CAIII

In the published article, the equal contribution note and symbol (†) were erroneously omitted. The author list was incorrectly displayed as “Xin-Ru Wang^1,2,3^, Shan-Shan Huang^1^, Meng Wang^1^, Jin-Hong Lin^4^, Jian-Tao Wang^1^, Jiao-Qi Ren^1^, Cheng-Feng He^2^, Wen-Jiao Xue^3^, Yin Wang^1^, Xue-Chun Wang^1^, Yan-Li Zhang^1^, Ji-Chang Xiao^4*^, Jing-Chun Guo^2*^ and Hou-Guang Zhou^1*^”.

The corrected author list appears below, with Xin-Ru Wang, Shan-Shan Huang and Meng Wang indicated as having equal contribution with the symbol “†”.

“Xin-Ru Wang^1,2,3†^, Shan-Shan Huang^1†^, Meng Wang^1†^, Jin-Hong Lin^4^, Jian-Tao Wang^1^, Jiao-Qi Ren^1^, Cheng-Feng He^2^, Wen-Jiao Xue^3^, Yin Wang^1^, Xue-Chun Wang^1^, Yan-Li Zhang^1^, Ji-Chang Xiao^4*^, Jing-Chun Guo^2*^ and Hou-Guang Zhou^1*^”.

^†^These authors have contributed equally to this work

The original version of this article has been updated.

