# Supplementary material for: S-9-PAHSA ameliorates cognitive decline in a type 2 diabetes mouse model by inhibiting oxidative stress and apoptosis via CAIII modulation
Source: Front Mol Neurosci. 2025 Aug 8;18:1617543. doi: 10.3389/fnmol.2025.1617543 (PMC12370719; doi:10.3389/fnmol.2025.1617543)
Supplement: Supplementary file 1 [file Data_Sheet_1.docx]

Supplementary Material

# 1 Supplementary Figures

# Supplementary Figure 1. Experimental design of this study.

# Supplementary Figure 2. SP could mitigate the CAⅢ reduction caused by diabetic models in vivo and in vitro. (A, B). Western blotting and quantification of CAⅢ protein in mice of ND, HFD, and SP-treated groups, n=5; (C, D). Western blotting and quantification of CAⅢ protein in PC12 cells exposed to standard medium (Con), high-glucose and high-fat medium (100 mM glucose + 200 μM palmitic acid, G100F200), G100F200 with DMSO, or G100F200 with 60 μM SP for 24 hours, n=5. Data are presented as mean ± SEM. *P<0.05, **P<0.01, ***P<0.001, ****P<0.0001.

**Supplementary Figure 3.** **CAⅢ gene knockdown in T2DM mice.** CAⅢ gene expression in hippocampus **(A)**, cortex **(B),** and brain **(C)** post-control or CAⅢ shRNA AAV injection, n=3; Body weight **(D)** and fasting glucose **(E)** before and after AAV injection in ND (n=15), HFD (n=15), HFD+AAV-Con (n=30), HFD+AAV-CAⅢ sh (n=30) groups. Data are presented as mean ± SEM. *P<0.05, **P<0.01, ***P<0.001, ****P<0.0001.

**Supplementary Figure 4**. **The efficiency of CAⅢ knockdown in PC12 cells.** **(A-B, D-E).** Western blotting and quantification of CAⅢ protein in CAⅢ sh, CAⅢ OE, and NC PC12 cells, n=5; CAⅢ mRNA levels in CAⅢ sh **(C)** and CAⅢ OE **(F)** cells, n=3. Data are presented as mean ± SEM. *P<0.05, **P<0.01, ***P<0.001, ****P<0.0001.

**Supplementary Figure 5. Immunofluorescence staining of TMRE to detect the effect of SP on mitochondrial membrane potential in PC12 cells.** Cells were treated for 24 hours with standard medium (Con), high-glucose and high-fat medium (100 mM glucose + 200 μM palmitic acid, G100F200), G100F200 with DMSO, or G100F200 with 60 μM SP. TMRE staining images for NC cells **(A)** and CAⅢ sh cells **(C)**; Corresponding fluorescence intensity quantification for NC cells **(B)** and CAⅢ sh cells **(D)**, n=5. Data are presented as mean ± SEM. *P<0.05, **P<0.01, ***P<0.001, ****P<0.0001.

**Supplementary Figure 6. Differential gene expressions (DEGs) in NC cells and CAⅢ cells treated with DMSO or SP. (A, B)** Volcano plots showing DEGs between DMSO and SP treatments in NC **(A)** and CAIII sh cells **(B)**, n=3; **(C, D)** Heatmaps of 13 DEGs in NC cells between the DMSO and SP groups in NC **(C)** and CAIII sh cells **(D)**, n=3. Rows represent individual cell samples, and columns correspond to genes. Color intensity reflects relative expression levels, with red indicating high and blue indicating low expression. **(E).** UpSet plot showing the intersection of DEGs across multiple comparisons. Each bar on the top represents the number of DEGs shared among the indicated sets, as shown by the connected dots below. Vertical bars denote the size of each intersection, while horizontal bars indicate the total number of DEGs in each group.
